# A fault-intrusion-tolerant system and deadline-aware algorithm for scheduling scientific workflow in the cloud

**DOI:** 10.7717/peerj-cs.747

**Published:** 2021-11-02

**Authors:** Mazen Farid, Rohaya Latip, Masnida Hussin, Nor Asilah Wati Abdul Hamid

**Affiliations:** 1 Department of Communication Technology and Networks, Universiti Putra Malaysia, Selangor, Serdang, Malaysia; 2 Faculty of Education-Saber, University of Aden, Saber, Aden, Yemen; 3 Institute for Mathematical Research (INSPEM), Universiti Putra Malaysia (UPM), Selangor, Serdang, Malaysia

**Keywords:** Cloud computing, Fault tolerance, Intrusion tolerance, Reliability, Scheduling scientific workflow

## Abstract

**Background:**

Recent technological developments have enabled the execution of more scientific solutions on cloud platforms. Cloud-based scientific workflows are subject to various risks, such as security breaches and unauthorized access to resources. By attacking side channels or virtual machines, attackers may destroy servers, causing interruption and delay or incorrect output. Although cloud-based scientific workflows are often used for vital computational-intensive tasks, their failure can come at a great cost.

**Methodology:**

To increase workflow reliability, we propose the Fault and Intrusion-tolerant Workflow Scheduling algorithm (FITSW). The proposed workflow system uses task executors consisting of many virtual machines to carry out workflow tasks. FITSW duplicates each sub-task three times, uses an intermediate data decision-making mechanism, and then employs a deadline partitioning method to determine sub-deadlines for each sub-task. This way, dynamism is achieved in task scheduling using the resource flow. The proposed technique generates or recycles task executors, keeps the workflow clean, and improves efficiency. Experiments were conducted on WorkflowSim to evaluate the effectiveness of FITSW using metrics such as task completion rate, success rate and completion time.

**Results:**

The results show that FITSW not only raises the success rate by about 12%, it also improves the task completion rate by 6.2% and minimizes the completion time by about 15.6% in comparison with intrusion tolerant scientific workflow ITSW system.

## Introduction

Cloud computing technology has become one of the most popular systems for providing end-users with computing services. An important feature of this paradigm is that the resources given can be accessed as a utility where consumers can pay for the services they use (Aslam et al., 2017; Chang & Wills, 2016; Farid et al., 2020; Ferdaus et al., 2017; Sun et al., 2018). In order to operate cloud-based applications in an economically-efficient and scalable way, it is highly favorable to deploy large virtual machines (VMs) (Ala’Anzy & Othman, 2019; Ghazouani & Slimani, 2017; Rao et al., 2013).

Scientific computing involves several interdependent intermediate data and subtasks which can be built up by different organizations (Yuan et al., 2010). To encourage the automation of complex scientific computational processes, scientific workflows were created (Rodriguez & Buyya, 2017). Scientific workflows are handled, controlled and executed by scientific workflow systems (Lin et al., 2009) derived from grid computing. With the advancement in cloud computing systems, several researchers are now developing cloud-based scientific workflow systems (Zhao et al., 2014).

Although cloud-based scientific workflow systems have several merits, cloud platforms are prone to malfunctions due to their increased functionality and complexity (Pezoa, Dhakal & Hayat, 2010; Yao et al., 2016). Such defects may have a detrimental impact on the performance of submitted tasks. This is because a system’s performance is not only evaluated by the correctness of the measurement results, but also by the time of its availability (Qin & Jiang, 2006).

Failure often occurs in the components of a system as the operation fails when a machine bears many loads (Gupta & Gupta, 2020). A task failure is a situation where the machine cannot complete the task within a deadline or when the machine ceases to process tasks due to network, memory, or system bugs. As a result of a delay in the completion of one of the tasks caused by a fault, several tasks on other resources may be delayed. To cater to this, many strategies have been proposed. In this paper, we develop a scientific workflow model for fault-intrusion tolerance. A deadline partitioning method determines the completion time for each sub-task.

Cloud platforms follow a multi-tenant coexistence service paradigm. As such, different tenants share the same physical facility using virtualization technologies. The implementation of this model creates flexible control of resources; however, it is associated with risks. There are several vulnerabilities in the virtualized world; an example is VM escape vulnerability (Wu et al., 2017). An intruder splits the logical boundaries into side channels and targets members in the same organization (Zhang et al., 2014). After the intruder has controlled the entire virtual environment with certain vulnerabilities, he controls the VMs of all tenants (Szefer et al., 2011).

Other threats in the cloud include co-residential attacks (Atya et al., 2017), side-channel attacks (Wang et al., 2016; Zhang et al., 2014), and VM escape attacks (Wu et al., 2017). A large number of subtasks and intermediate data contained in scientific workflows can easily be targeted by attackers. In addition to researchers’ efforts to address threats in the cloud, we propose a fault-intrusion tolerant system and deadline-aware resource provisioning algorithm to protect workflows in clouds. The dynamic task scheduling strategy, based on resource circulation, eliminates latent threats. By regularly deploying and reclaiming VMs, the proposed approach cleans up task executors. The performance of FITSW was tested using WorkflowSim.

Realizing intrusion tolerance is much harder than fault tolerance because it should consider both accidental and malicious faults. Our objective is to achieve the intrusion tolerance of scientific workflows scheduling process.

Our contributions in this study are summarized as follows:

1. We propose a fault and intrusion-tolerant mechanism for scientific workflows (FITSW) by considering the effects of accidental and malicious faults on cloud-based scientific workflows.
2. We develop task executors with various heterogeneous VMs having multiple operating systems.
3. We present a decision-making mechanism that tracks and evaluates the confidence of the intermediate data between sub-tasks during execution.
4. To eliminate latent risks, we suggest a dynamic task scheduling strategy based on recycling resource. FITSW keeps task executors clean by installing and reclaiming virtual machines on a regular basis.

The remainder of this article is organized as follows: “Literature Review” reviews related works. FITSW’s principle and threat model are introduced in “Principle of FITSW and Threat Model”. “Proposed Scheme” outlines the new scheme. In “Experiments and Results”, the experiments and results are discussed, followed by the “Conclusion” that concludes this article.

## Literature review

Many fault-tolerant algorithms have been proposed in recent decades to reduce the adverse effects of faults in distributed systems. Javadi et al. (2011) investigated how failures due to faults can be handled in complex infrastructures. However, the model often involves tracing data failures related to a particular objective, which can be very challenging. Jhawar & Piuri (2012) suggested a new dimension in which required fault tolerance properties can be obtained by a third party from applications deployed in cloud systems. For ordinary users, however, it is difficult to choose the appropriate third party. Zheng et al. (2012) and Qiu et al. (2014) suggested a ranking method in which all components of the cloud were categorized according to invocation structures and invocation frequencies. Using an optimal algorithm based on the ranking results, the fault-tolerant strategies for the various components were computed. However, the exact ranking is difficult as it requires a thorough understanding of the behavior of the target infrastructure as well as long-term trace data of the specific system.

Related to our work which focuses primarily on the study of fault and intrusion tolerance in scientific workflow scheduling, Yao et al. (2016) proposed a workflow scheduling algorithm inspired by the immune system. This algorithm can prevent cloud-based scientific workflow disruptions (due to the failure of resources) to protect scientific workflow sub-tasks. To ensure that cloud services are continuously available to defend against security threats, a cloud resource management self-protection solution was introduced by Gill & Buyya (2018). Centered on the master-slave theorem, Ding, Yao & Hao (2017) developed a fault-tolerant scheduling algorithm. For each sub-task, two replicas (one master and one slave replica) are generated and allocated to individual VMs using this algorithm. The ICFWS algorithm, proposed by Yao, Ding & Hao (2017) divides the general workflow deadline into sub-task sub-deadlines. Then, based on the assigned sub-deadlines, each scientific workflow task selects an acceptable fault-tolerant strategy using task redundancy and rescheduling strategies.

Cloud services are priced dynamically and are referred to as spot instances of the VMs. Spot cases are cheaper than the VMs offered by the static price scheme. Therefore, Poola, Ramamohanarao & Buyya (2016) suggested the use of spot instances to execute scientific workflows in order to minimize cost. Li et al. (2016) quantified security for cloud services and thoroughly analyzed the risk rates of scientific workflows. The researchers then created the Security and Cost Aware Scheduling (SCAS) scheme to reduce costs when risk rates are small and deadlines are tight. The cloud-based scientific protective problem in the workflow was formulated as a two-person zero-sum problem by Wang et al. (2020), who suggested the CLOSURE algorithm to confuse adversaries. Nevertheless, if an attacker manages to access a VM and tampers with the scientific workflow to generate an incorrect output, this problem cannot be solved effectively by either of the above works.

Some studies analyze failures to ensure that the workflow is performed successfully even if resources fail. Secret data and sensitive computation also require scientific workflows (Wang et al., 2019b). This motivates the need for secure execution of scientific workflows. In this context (Chen et al., 2017) used the scientific workflow’s slack times to encrypt intermediate data. The encryption algorithm was combined with the task scheduling algorithm to ensure the scientific workflow’s confidentiality and reduce cost and time. Liu et al. (2014) suggest a security aware intermediate data placement strategy to ensure that intermediate data is secured in three ways: integrity, confidentiality, and privileged access.

In order to increase the availability, confidentiality and integrity of the data of scientific workflow (Wang et al., 2019b) used different hash functions, encryption algorithms, and erasure codes while considering scientific workflow deadlines. Teylo et al. (2017) studied the scheduling of scientific workflow and the intermediate data allocation. They modeled them as part of an integrated planning challenge to reduce intermediate data transmission in a cloud network. A hybrid evolutionary algorithm known as HEA-TaSDAP was created to optimize task planning, intermediate data allocation strategies and task scheduling. TryXy was created by Nepal et al. (2017) and it offers stable scientific workflow storage facilities.

In Wang et al. (2019a), the ITSW designer used a mission replication and voting system to prevent attackers from modifying the results of scientific workflows. But ITSW ignores the delay that can happen because of accidental (Yao, Ding & Hao, 2017) or malicious attacks (Bhattarai et al., 2014, 2015) which could lead to an increase in the makespan of the entire workflow. In order to determine sub-deadlines for each sub mission (Wang et al., 2021) proposed the INHIBITOR to determine sub-deadlines for each submission by using the deadline partitioning process. The system for the provision of elastic resources is structured to maximize efficiency and reduce costs, based on these sub-deadlines. This paper deploys the Task-VM mapping framework and elastic resource provisioning mechanism to enhance ITSW intrusion tolerance. Our aim is to develop a FITSW workflow scheduling algorithm that will improve the system’s fault and intrusion tolerance, taking into account the delay due to accidental failures or intrusion attacks.

### Scientific workflow security issues in the cloud

It is quite difficult to address the security issues of scientific workflows in clouds due to their unique features. Many scientific workflows are computational intensive (Li et al., 2016), thus, they need VMs. These VMs can be targeted easily by attackers (Narayana & Pasupuleti, 2018). Executing scientific workflows is also time-consuming (Yuan et al., 2010). This provides enough time for attackers to make an intrusion. A scientific workflow is typically a form of Directed Acyclic Graph (DAG) that is extremely vulnerable to attacks, as intermediate errors are inherited in the final result (Wang et al., 2018). In addition, the intermediate scientific workflow data also contain sensitive data in some scientific fields. If this data is hacked, users will suffer severe damage (Wang et al., 2019b). Adversaries can compromise workflow execution in several ways.

1. The attackers can access and force the VMs that run workflows to go offline.
2. The intruder can gain access to intermediate data without tempering or altering it, rather he delays the finish time of executing sub-tasks (Yu et al., 2017). Attacks such as jamming attacks, sniffer attacks, worm propagation, and resource-depletion denial-of-service (Bhattarai et al., 2014, 2015) could be launched to disable the links by congesting the network or monitoring network data flow (Bhattarai et al., 2015; Yu et al., 2017).
3. In some cases, the purpose of the adversaries is to alter the workflow result rather than interrupt the workflow. This can be achieved through intermediate data workflow manipulation and execution software.
4. The adversaries can also steal the workflow data after breaching the VMs or build a back door for the next attack.

The fault tolerance system generally protects against the first and second types of attacks. However, it cannot prevent the remaining two types. To develop a Fault-Intrusion-tolerant system capable of effectively defending against these four types of threats. There are four major challenges to overcome.

1. To check that each sub-task can be performed without any VM failures, the systems must be able to check the average earliest finish time of the virtual cluster using the sub-task sub-deadline.
2. Systems must be able to (i) assess if workflow’s sub-task results are right by checking the confidence of the intermediate data of all replicas and (ii) correct altered outputs to protect the system against the third form of attack by re-executing the current task.
3. The system should be strong enough to withstand the fourth form of attack by removing latent threats and cleaning up executors using resource recycling technique.
4. Instead of security, the efficiency of the system is also very important. Therefore, the system’s fault-intrusion-tolerant mechanism should not negatively impact workflow efficiency according to the proposed decision mechanism.

## Principle of fitsw and threat model

### Principle of FITSW

The DAG is an example of a scientific workflow. It is expressed by *G* = (*T, D*), where *T* = {*t*_1_, *t*_2_, *…*, *t*_*n*_} represents a collection of subtasks for workflow and *D* is representing the intermediate data between sub-tasks. *d*_*i,j*_ ∈ *D* refers to intermediate data generated from subtask *t_i_* and used by subtask *t_j_*. every workflow sub-task *t*_*i*_ is measured in Million instructions (*MIi*) (Jhawar & Piuri, 2012). pred (*t*_*i*_) and succ (*t*_*i*_) represent the predecessors and successors of subtask *t*_*i*_, respectively. Sub-task *t*_*i*_ cannot begin in scientific workflows until all its predecessors are completed.

The workflow executor is one VM in several cloud-based scientific workflow systems. It ensures that every subtask workflow is completed with just one VM. This is highly risky as the adversaries will only jeopardize a single VM to destroy workflow execution. Therefore, a virtual cluster (Liu et al., 2018) is suggested for task execution. There are several VMs in the virtual cluster and these VMs run the same workflow subtask in parallel.

Three replicas of each workflow sub-task *t* are copied into FITSW: *t*^*first*^ (the first replica), *t*^*second*^ (the second replica) and *t*^*third*^ (the third replica), which are executed by three heterogeneous VMs. The heterogeneity of VMs is represented primarily by variations in operating systems. Linux VM, Windows VM and Solaris VM operate for each sub-task workflow. The decision mechanism in FITSW relies mainly on identical results and the sub-deadline of the sub-task. If the confidence of any sub-task is less than 1, the sub-task is re-executed.

### Principle of ITSW-RV

In ITSW-RV, we deploy a random virtual task execution cluster with different VM numbers (between 3–10). The number of replicas for each subtask must be the same for a random number of VMs with different operating systems. The number of similar results is increased in this situation and the ability to receive most of the results is also increased. Our experiment indicates that an increase in the number of VMs contributes to a reduction in the overall algorithm completion time.

### Task-VM mapping

Aside from the resource provisioning strategy, task-VM mapping is an essential step in workflow scheduling also (Rodriguez & Buyya, 2017). Various task-VM mapping relationships can be used to achieve various scheduling objectives, such as decreasing the cost of cloud scientific workflows (Zhou, He & Liu, 2016), maximizing the use of virtual machines (VMs) during the execution of scientific workflows(Lee et al., 2015), and reducing the makespan for a scientific workflow to complete (Jiang, Haihong & Song, 2017). In an actual production environment, the authors suggest in Sangaiah et al. (2019) an algorithm for scheduling tasks to be mapped into machines. Similarly, a subtask cannot begin execution before a VM has been allocated. As a result, before using the proposed task-VM mapping algorithm, we will first allocate VMs. Additionally, the system cannot use mapped VMs for the second and third replicas. Finally, for any sub-tasking replica, the HEFT (Topcuoglu, Hariri & Wu, 2002) algorithm selects the required VM. The steps taken are described below.

1. To perform tasks, the upward ranking approach is employed as shown in Eq. (1).
(1) $$ran{k_u}\left( {{t_i}} \right) = \overline {{\omega _i}} + ma{x_{{t_j} \in succ({t_i})}}\left\{ {{{{d_{i,j}}} \over {BW}} + ran{k_u}\left( {{t_j}} \right)} \right\}$$
  The average time available for the VMs to perform *t*_*i*_ is *ω*_*i*_. By crossing the task graph upward, the rank is recurrently calculated, so it is called upward rank. The upward rank value for the last subtask *t*_*last*_ is equivalent to Eq. (2).
(2) $$ran{k_u}\left( {{t_{last}}} \right) = \overline {{\omega _{last}}}$$
2. Every sub-task is sorted by a non-increasing *rank_u_* value in a scheduling list.
3. A subtask is chosen in the schedule list and copied into three replicas.
4. For all replicas, $t_i^{first}$, $t_i^{second}$ and $t_i^{third}$, each Earliest Finish Time (EFT) value on VM *vm*_*j*_ is calculated using Eqs. (3) and (4).

Only direct predecessors of a subtask are needed to decide the earliest start time of the three replicas.

(3) $$\eqalign{EST( {t_i^x,v{m_j}} ) = max\bigg\{ {read{y_j},ma{x_{{t_m} \in pred( {{t_i}} )}}\bigg\{ {ma{x_{y \in \{ {first,second,third} \}}}\bigg\{ {EFT( {t_m^y} ) + {{{d_{m,i}}} \over {BW}}} \bigg\}} \bigg\}} \bigg\}, \cr x \in \{ {first,second,third} \}}$$

(4) $$EFT\left( {t_i^x,v{m_j}} \right) = {{M{I_i}} \over {vm{p_j}}} + EST\left( {t_i^x,v{m_j}} \right),x \in \left\{ {first,second,third} \right\}$$

*ready*_*j*_ shows the earliest time at which *vm*_*j*_ is ready to execute sub-tasks, *EFT*( $t_m^x$) denotes the earliest finish time of $t_m^x$, The size of each sub-task *t*_*i*_ is measured in *MI*_*i*_ (Jhawar & Piuri, 2012). *vmp*_*j*_ represents the processing power of *vm*_*j*_ and it is measured in Million instructions per second *MIPS*.

As predicted, a delay with no malicious connections is less than a delay with malicious connections (Bhattarai et al., 2014), the finish time of the sub-task must be less than the sub-deadline. Otherwise, intruders would have completed their objectives before the response party performs critical path analysis. In our model, we use a task executor that contains three *VMs* with different operating systems to execute individual sub-task. Hence, to determine the *EFT* of each sub-task and compare it with the sub-deadline, we calculate the average *EFT* of all replicas for each subtask by the following equation.

(5) $$AEFT\left( {{t_i}} \right) = {{\sum\limits_{x = first}^{third} E FT\left( {t_i^x,v{m_j}} \right)} \over {no.\ of\ vms \ in\ virtual\;cluster}}$$

### Proposed Scheme

**Figure 1:**
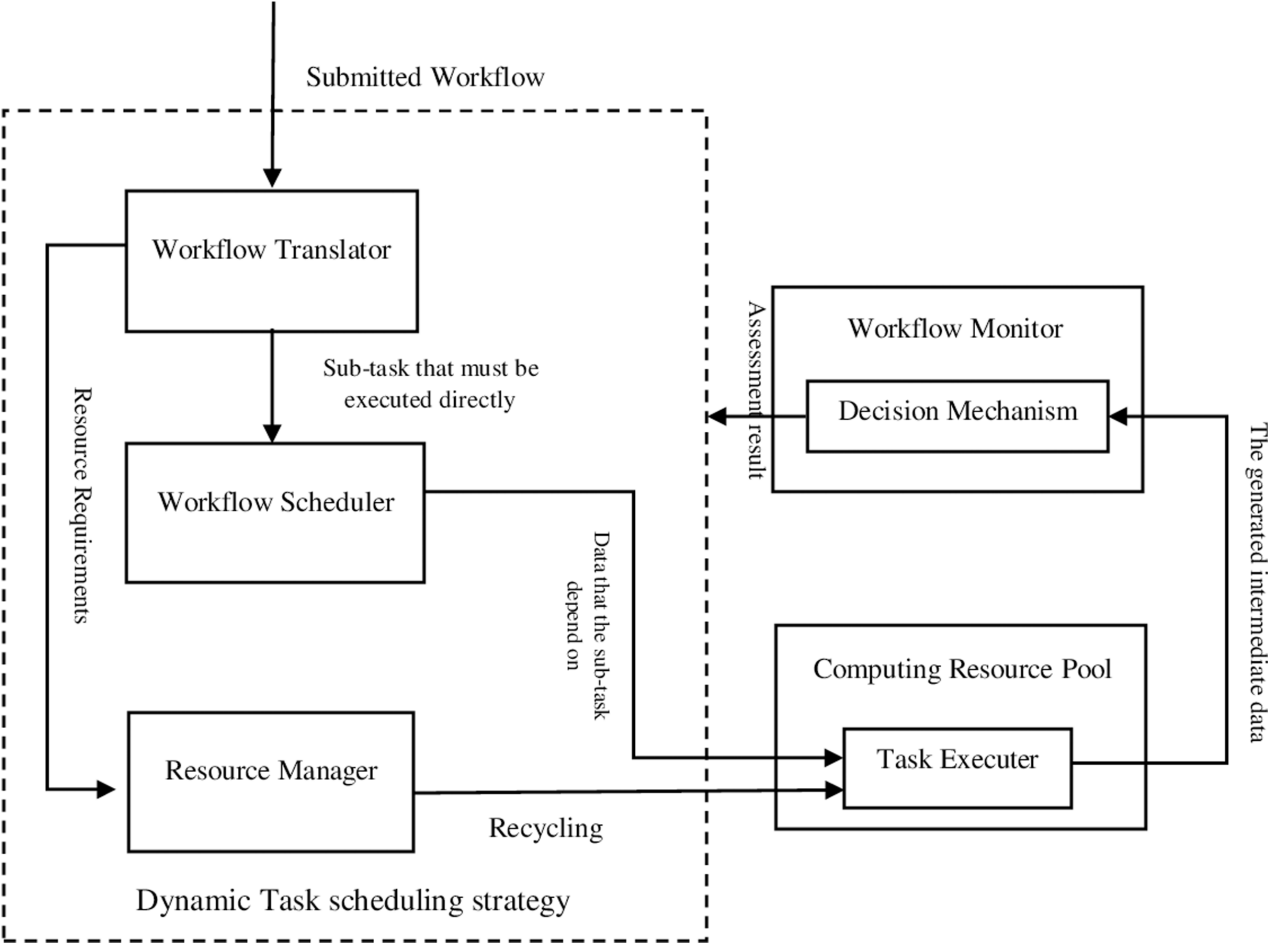
The proposed FITSW system.

The outline of the proposed FITSW system is illustrated in Fig. 1. We recommend that multiple heterogeneous *VMs* should be used as task executors to improve the workflow execution fault tolerance. The proposed decision mechanism verifies and evaluates the confidentiality of intermediate data by the executors. It also checks the earliest finish time of each subtask. According to the evaluation of the proposed decision mechanism, a dynamic task scheduling strategy is applied based on resource circulation. It decides whether to implement or recycle task executors. This approach removes inherent threats and cleans the environment for executing scientific workflows.

### Proposed decision mechanism

Attackers mainly aim at controlling the outcome of a workflow rather than terminating it. This could lead to a false workflow output. If the task executer is compromised by this type of attack, the sub-tasks will have multiple results. A decision mechanism to turn multiple inputs into one output can be applied in order to avoid this threat, thereby protecting against inconsistent system states. The time to finish the sub-task is therefore not the same for each virtual machine.

**Figure 2:**
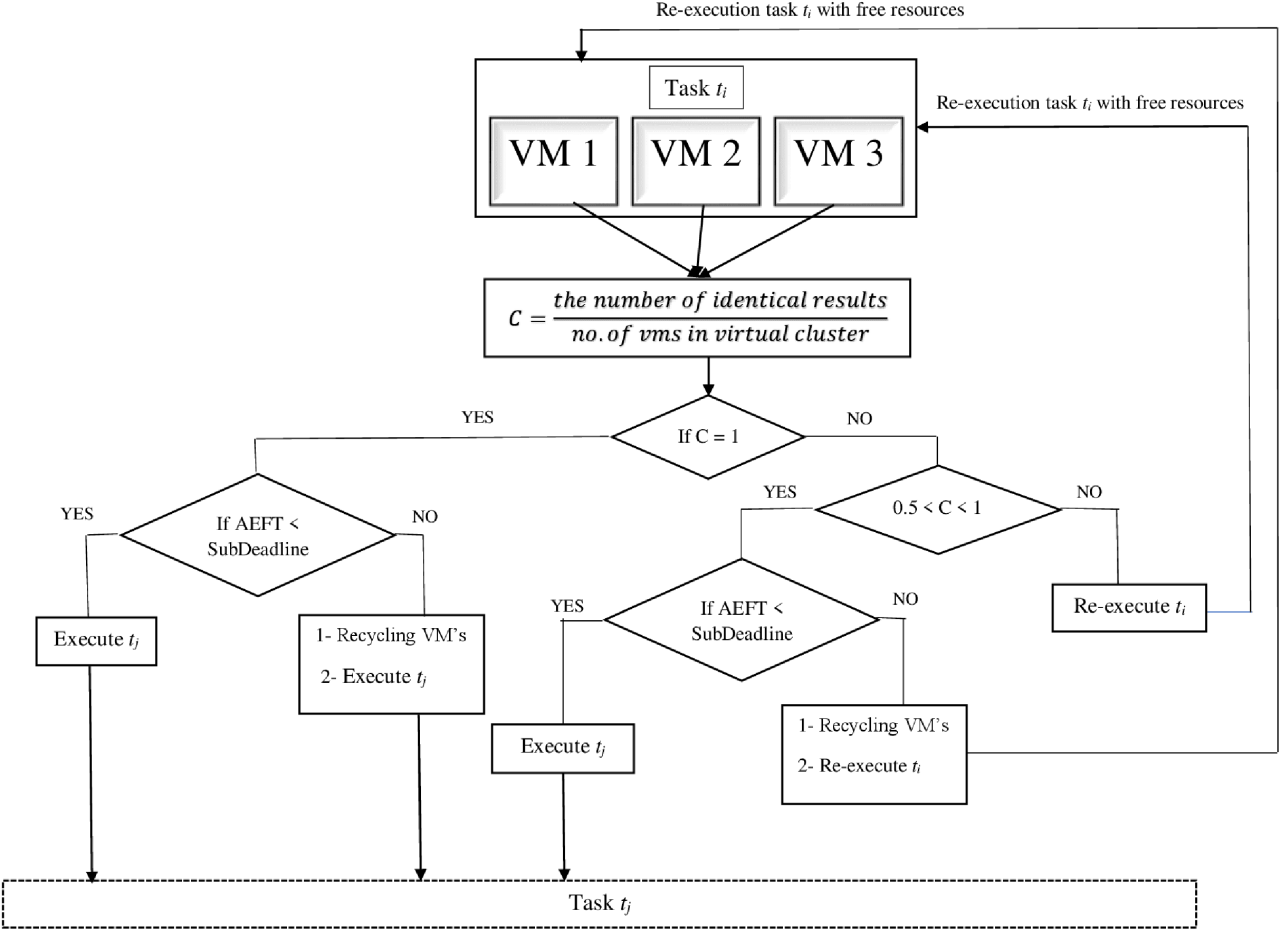
Decision mechanism.

Using *t*_*f*_ and *t*_*l*_ to denote the states within the system (*i.e*., the time when the first results and the last results are produced, respectively), if the decision mechanism only generates the outcome after all VMs have obtained the results, the execution time of each workflow sub-task is increased by *t*_*l*_ *− t*_*f*_. In order to collect the results from all VMs, the time of execution of each sub-task of the workflow will increase by *t*_*l*_ *− t*_*f*_. The proposed decision mechanism converts the intermediate data produced to an MD5 value for verification. Fig. 2 shows its principle.

We presume that there are sub-tasks *T*_*i*_ and *T*_*j*_, and the sub-task *T*_*j*_ relies on the sub-task *T*_*i*_ to generate intermediate data. Confidence *C* for each intermediate data is determined by Eq. (6) using the number of identical results.

(6) $$C = {{the\;number\;of\;identical\;results} \over {no.\ of\ vms \ in\;virtual\;cluster}}$$

The decision model can assume five cases during the collection of outputs.

Case 1: *C* = 1 and *AEFT* < sub-deadline; the decision module will execute sub-task *T*_*j*_.

Case 2: *C* = 1 and *AEFT* > sub-deadline. In this case, two actions will be taken by the decision module. First, recycling all the VMs that executing sub-task *T*_*i*_ because the *AEFT* is more than sub-deadline. This delay might result from some threats. Second, executing sub-task *T*_*j*_ because the confidence is 1.

Case 3: 0.5 < *C* < 1 and *AEFT* < sub-deadline. In this case *T*_*j*_ is executing because the majority of results are identical.

Case 4: 0.5 < *C* < 1 and *AEFT* > sub-deadline. In this case, two actions will also be taken by the decision module. First, recycling all the VMs executing sub-task *T*_*i*_ because the *AEFT* exceeds the sub-deadline. Second, re-executing *T*_*i*_ because the confidence is less than one.

Case 5: *C* ≤ 0.5; sub-task *T*_*i*_ will be re-executed instantly with the available resources.

In case 2, the confidence is 1 and *AEFT* exceeds the sub-deadline of sub-task. We assume that the increase in *AEFT* could happen due to some accidental faults resulting in this delay.

In case 3, the confidence is less than 1 and *AEFT* of the virtual cluster is less than the sub-deadline. Here, we assume that this might occur as a—result of accidental faults because there is no delay during the process.

In case 4, the confidence is less than 1 and the *AEFT* of the virtual cluster is more than the sub-task sub-deadline. In this case, we assume this low confidence level is due to threat and delay. So, the significance of the sub-task re-execution is significantly high.

In case 5, the confidence is less than or equal to 0.5; thus, the current sub-task must be re-executed because of the low confidence level of the intermediate data.

Without reducing efficiency, the suggested decision mechanism will enhance the credibility of effective workflow execution.

### Fault and intrusion-tolerant workflow scheduling algorithm

To use the FITSW algorithm, first determine the sub-deadline for each sub-task using a deadline partitioning method. An elastic resource provisioning scheme is built based on these sub-deadlines, and a task-VM mapping technique is used to improve performance.

#### Deadline partitioning

To ensure that FITSW meets the workflow deadline, we use the approach of dividing the deadline (Cao et al., 2019; Wang et al., 2021). This divides the deadline given by the user into sub-deadlines for individual scientific workflow sub-tasks. First, this approach uses Eq. (7) to measure upward rank for each subtask,

(7) $$ran{k_u}\left( {{t_i}} \right) = ma{x_{{t_j} \in succ\left( {{t_i}} \right)}}\left( {{\gamma _j}.{{size\left( {{d_{i,j}}} \right)} \over {BW}} + ran{k_u}\left( {{t_j}} \right)} \right) + \omega _{\rm{i}}^{\rm{*}}$$

$\omega _{\rm{i}}^{\rm{*}}$ shows the time *t*_*i*_ of the fastest VM. In Eq. (8), ${\gamma _j}$ is used to determine communication costs size ( ${d_{i,j}}$)/BW.

(8) $${\gamma _j} = \left\{ {\matrix{0, & 1 - {\theta ^{ - {k_j}}} \\ 1 & otherwise \\ } } \right.$$

*θ* is a parameter that is predefined to be greater than 1. In our experiments, we set the value of *θ* to 1.2. *K*_*j*_ denotes the ratio execution time *t*_*j*_ to communication time, which can be determined by Eq. (9).

(9) $${k_j} = {{\omega _{\rm{i}}^{\rm{*}}.{\rm{BW}}} \over {{d_{i,j}}}}$$

Then, the sub-deadline of sub-task *t*_*i*_, *i.e*., *subD(t*_*i*_*)* can be calculated by Eq. (10).

(10) $$subD\left( {{t_i}} \right) = {{ran{k_u}\left( {{t_{init}}} \right) - ran{k_u}\left( {{t_i}} \right) + \omega _{\rm{i}}^{\rm{*}}} \over {ran{k_u}\left( {{t_{init}}} \right)}}.D$$

where *t*_*init*_ denotes the workflow initial sub-task and *D* represents the deadline provided by the user.

### Dynamic task scheduling strategy based on resource circulation

The workflow environment in a traditional cloud workflow system is static and unchanged. As a result, attackers will launch backdoor attacks and leak information. If attackers gain access to VMs, they can maintain control over them for a prolonged period of time. A dynamic task scheduling approach based on resource circulation is proposed as a solution to this problem. The method recycles VMs that perform sub-tasks and creates new VMs without raising users’ sub-task resource requirements. The dynamic task scheduling method is implemented using the decision process depicted in Algorithm 1.

**Algorithm 1:** FITSW.

**BEGIN**
1. ${\bf{Input}}\ K(The\ number\ of\ VMs\ included\;in\;vrtual\;cluster)$
2. $ran{k_u}\left( {{t_i}} \right) = \overline {{\omega _i}} + ma{x_{{t_j} \in succ({t_i})}}\left\{ {{{{d_{i,j}}} \over {BW}} + ran{k_u}\left( {{t_j}} \right)} \right\};$
// $Calculate\;the\;upword\;ranking\ of\ sub\_task\ {T_i}\ according\ to$ (1)
3. $subD\left( {{t_i}} \right) = {{ran{k_u}\left( {{t_{init}}} \right) - ran{k_u}\left( {{t_i}} \right) + \omega _{\rm{i}}^{\rm{*}}} \over {ran{k_u}\left( {{t_{init}}} \right)}}.D$;
// $Calculate\ the\ sub\_deadline\ of\ sub\_task\ {T_i}\ according\ to $ (10);
4. $Recieving\ sub\_task\ {T_i}\ replicas\;execution\;results\;from\;virtual\;clusters$;
5. $confidence = {{the\ number\;of\;identical\;results} \over {no.of\ vms\ in\ virtual\;cluster}};$
// $Calulate\;the\;confidance\;of\;intermediate\ data\ of\ sub\_task\ {T_i}\ according\ to$ (6);
6. $AEFT\left( {{t_i}} \right) = {{\sum\limits_{x = first}^{third} E FT\left( {t_i^x,v{m_j}} \right)} \over {no.of\ vms\ in\ virtual\;cluster}};$
// $Calculate\ the\ AEFT\ of\ virtual\;cluster\;according\ to$ (5);
7. ${\bf{if}}(confidence = 1\ and\ AEFT \lt sub\_deadline)$
8. $generating\ new\ VMs\ to\ executing\ sub\_task\ {T_j};$
9. ${\bf{if}}(confidence = 1\ and\ AEFT \gt sub\_deadline)$
10. $Recycling\ all\ the\ VMs\ executing\ sub\_task\ {T_i};$
11. $generating\ new\ VMs\ to\ executing\ sub\_task{T_j};$
12. ${\bf{if}}((0.5 \lt confidence \lt 1)\ and\ (AEFT \lt sub\_deadline))$
13. $generating\ new\ VMs\ to\ executing\ sub\_task\ {T_j};$
14. ${\bf{if}}((0.5 \lt confidence \lt 1)\ and\ (AEFT \gt sub\_deadline))$
15. $Recycling\ all\ the\ VMs\ executing\ sub\_task\ {T_i};$
16. $Re\_execute\ sub\_task\ {T_i}$;
17. ${\bf{if}}(confidence \le 0.5)$
18. $Re\_execute\ sub\_task\ {T_i}$;
**END**

The proposed task scheduling model can be used for VM cleaning. If viruses infect any VM during the workflow, the proposed job/task scheduling strategy would clear the affected VM. In addition, the dynamic task scheduling technique will prevent attackers from sniffing into workflow data on a regular basis. With the dynamic task scheduling approach, the proposed framework would recycle VMs that had completed subtask execution. It will also prevent attackers from taking control of a virtual machine for an extended period of time.

## Experiments and results

### Experimental setting

**Figure 3:**
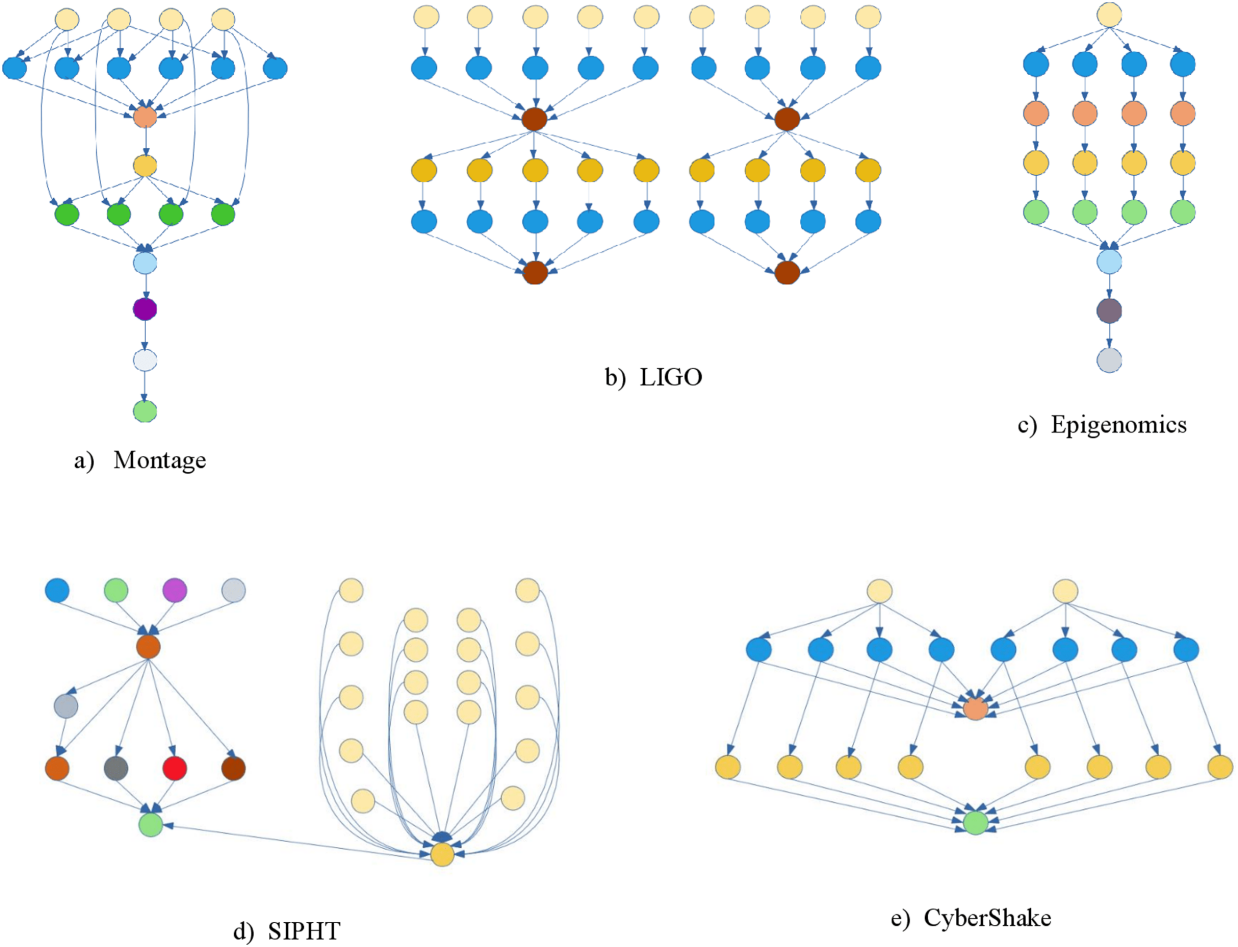
Some scientific workflows structures.

Experiments were performed using WorkflowSim (Chen & Deelman, 2012), an open-source cloud workflow simulation software, where the scientific workflow is represented in XML. The Montage, Epigenomics, CyberShake, Inspiral, and Sipht scientific workflows introduced by Pegasus (Deelman et al., 2015) were used for these experiments. The scientific workflow structures are provided in Fig. 3 while the parameters are given in Table 1. Three metrics were used to measure the performance of the algorithm:

**Table 1:** The parameters of different workflows.

Scientific workflow	Number of sub-task	Number of edges	Average data size (MB)	Average sub-task runtime (s)
CyberShake_1000	1,000	3,988	102.29	22.71
Epigenomics_997	997	3,228	388.59	3,858.67
Montage_1000	1,000	4,485	3.21	11.36
Inspiral_1000	1,000	3,248	8.90	227.25
Sipht_1000	1,000	3,528	5.00	179.05

1. Success Rate (SR) achieved after executing scientific workflows (when there are attacks): SR denotes the algorithm’s intrusion tolerance.
2. Workflow Makespan: This represents the algorithm’s completion time.
3. Task Completion Rate (*TCR*) (Yao, Ding & Hao, 2017): Calculated as shown in Eq. (11).

(11) $$TCR = {{N\ baseline} \over {N\ workflow}}$$

The HEFT workflow makespan is used as a baseline while *Nbaseline* denotes the number of sub-tasks performed at baseline time. *Nworkflow* reflects the number of workflow sub-tasks that were used in the test. The *TCR* demonstrates the algorithm’s efficiency.

To evaluate FITSW, three experiments are conducted with the following aims: (1) Evaluating FITSW’s *SR* with different numbers of VM’s attack (2) Evaluating FITSW’s *TCR*. (3) Evaluating FITSW system’s efficacy by calculating completion time without considering attacks. In this experiment, we compare FITSW with ITSW (Wang et al., 2019a) and ITSW-RV with a random number of VMs. For the final results presentation, average values are considered. Each test is carried out twenty-five times. The processing power of each VM is produced randomly during each experiment from 1,300 *MIPS* to 2,000 *MIPS*.

### Evaluating SR

The intrusion tolerance is quantified by *SR* in this experiment. The higher the algorithm’s *SR*, the greater the intrusion tolerance for the same number of compromised VMs.

(12) $$SR = {{number\;of\;simulation\;runs\ that\;successfully\;meet\;the\;deadline} \over {total\;number\;of\;simulation\ runs}}$$

First, we presume that the adversaries capture one VM, which generates an incorrect result for any sub-task assigned to this VM. The average *SR* of FITSW, ITSW, and ITSW-RV for different numbers of available VMs is determined for these purposes using five different scientific workflows. Tables 2 and 3 demonstrate the results.

**Table 2:** SR of FITSW, ITSW and ITSW-RV under the condition of one compromised VM.

Algorithms	The number of available VMs						
	30	50	100	150	200	250	300
ITSW	0.04	0.08	0.28	0.6	0.52	0.68	0.8
ITSW-RV	0.16	0.16	0.6	0.52	0.56	0.72	0.8
FITSW	0.16	0.24	0.76	0.64	0.72	0.92	1

**Table 3:** SR of FITSW, ITSW and ITSW-RV under the condition of two compromised VMs.

Algorithms	The number of available VMs						
	30	50	100	150	200	250	300
ITSW	0.04	0.08	0.4	0.48	0.40	0.64	0.76
ITSW-RV	0.08	0.08	0.56	0.56	0.52	0.64	0.8
FITSW	0.16	0.24	0.52	0.58	0.64	0.8	0.8

Second, as the number of compromised VMs increases, the FITSW and ITSW *SR*s decrease dramatically. However, ITSW exhibits a more obvious downward trend than FITSW. Also, the number of available VMs influences FITSW and ITSW. This is because the higher number of VMs available, the lower the chances that two compromised VMs will be assigned to two of the three sub-task replicas. In general, FITSW is more intrusion-tolerant than ITSW.

### Evaluating TCR of FITSW

**Figure 4:**
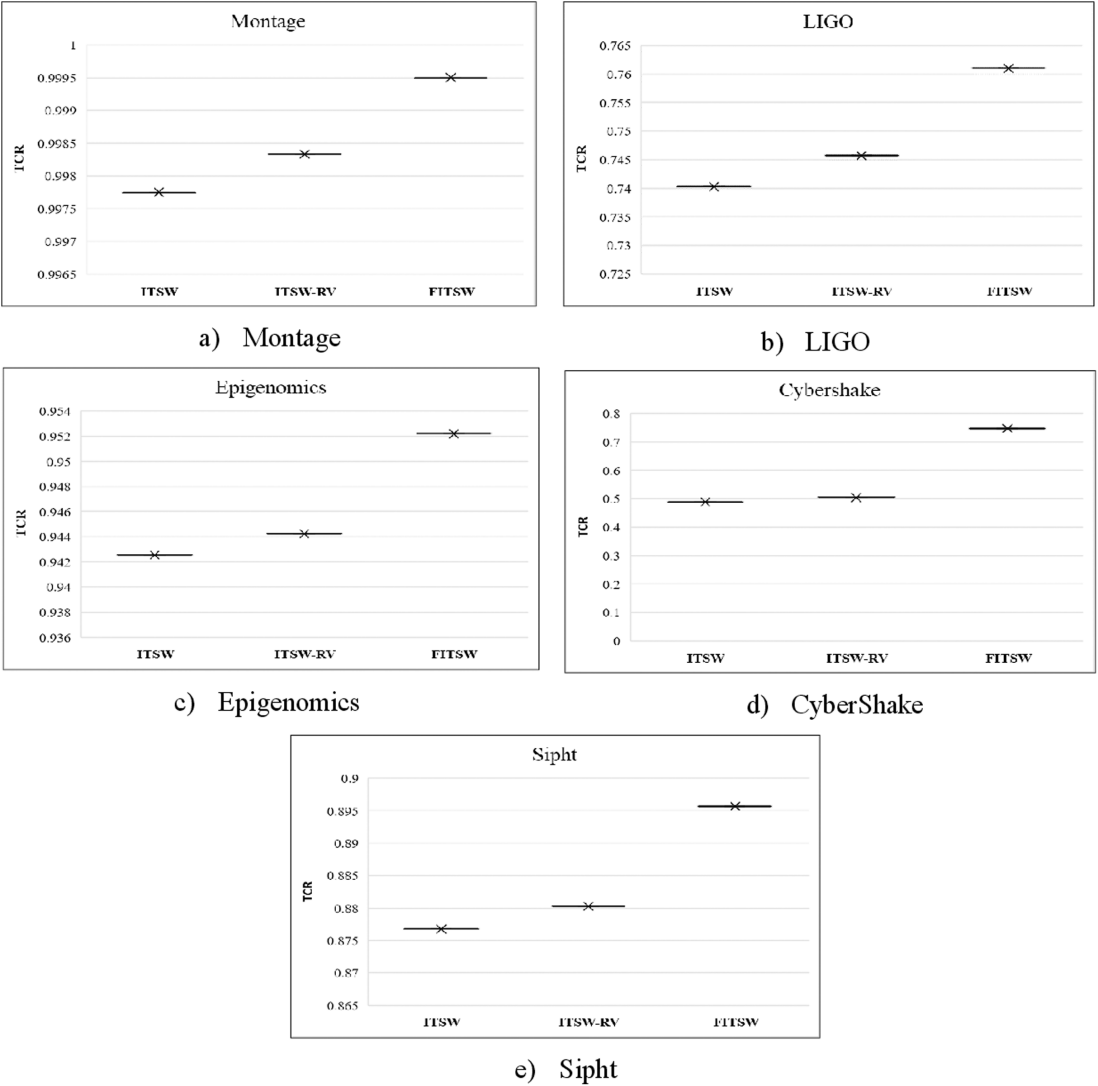
(A–E) The TCR of FITSW, ITSW and ITSW-RV.

Here, we test TCR with a random number of VMs for FITSW, ITSW and ITSW-RV. TCR is defined as the relationship between tasks completed and the total number of workflow tasks checked during the soft-deadline. It reflects the effectiveness of the task performed with respect to the compared algorithms. At first, there are 100 VMs available and the HEFT makespan is the baseline. The deadline for FITSW, ITSW and ITSW-RV is (1.2*Baseline). From the result shown in Fig. 4, the TCR of ITSW is lower than that of FITSW and ITSW-RV. This results because FITSW is not based on a backup mechanism and the number of VMs available is not enough to meet the execution requirements. To support cloud-based scientific workflows, ITSW-RV can increase its resource pool while FITSW and ITSW have a fixed number of VMs.

### Efficiency assessment of FITSW system

**Figure 5:**
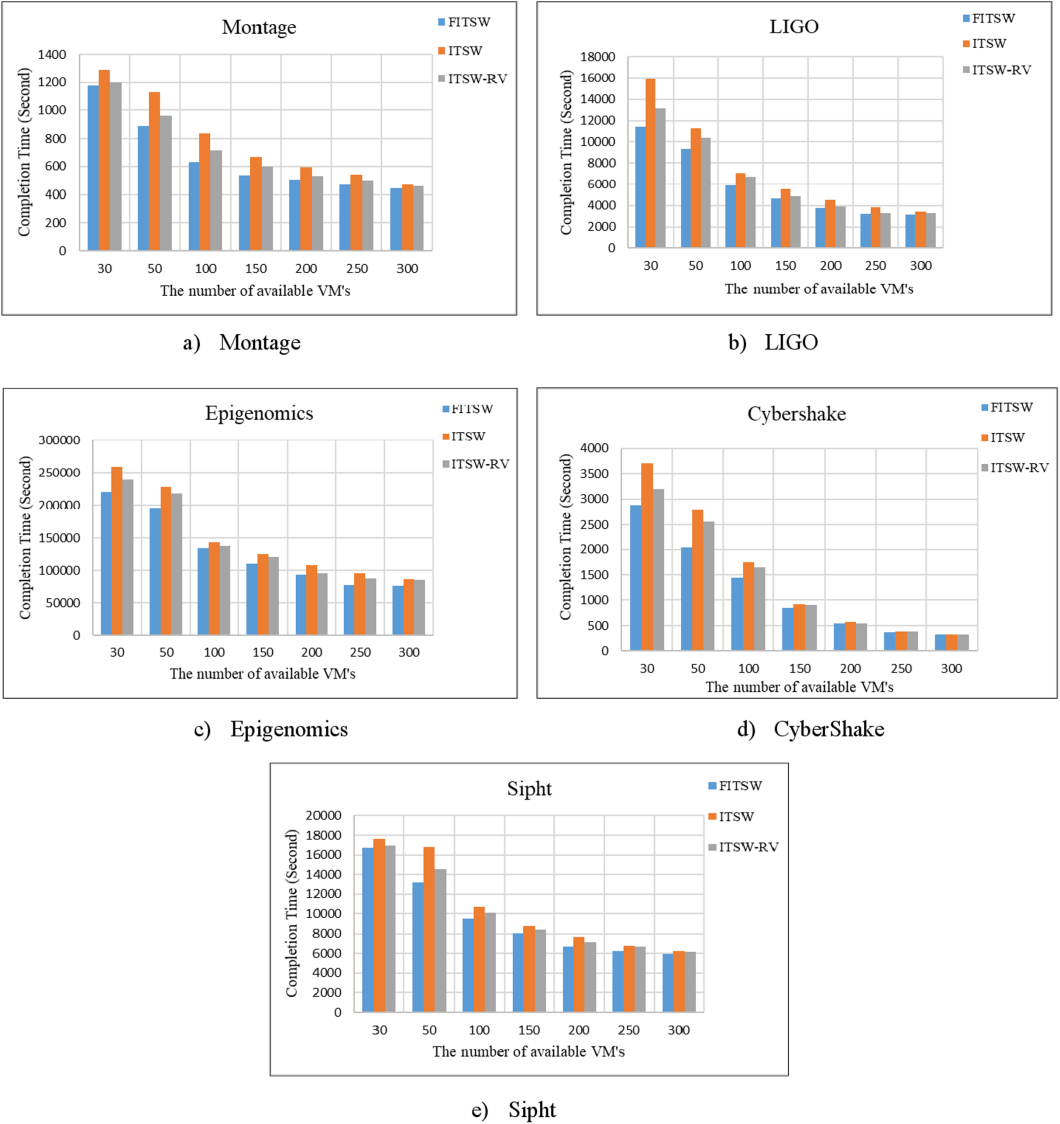
(A–E) The completion time of FITSW, ITSW and ITSW-RV.

First, without considering an attack, we checked the FITSW system’s efficiency. The number of VMs that are available is used as variables. FITSW, ITSW and ITSW-RV systems workflow completion time is shown in Fig. 5. From the figure, it is obvious that the completion time of FITSW system is smaller than ITSW and ITWS-RV. This can be traced to the fact that FITSW checks the confidence value and sub-deadline for each sub-task to decide on whether to re-execute the current sub-task or execute the next. ITSW and ITSW-RV will inevitably take more time waiting for results because of its intermediate data backup mechanism for temporary workflow. The completion time of the FITSW system decreased by around 15.6% on Montage, 19% on Inspiral, 18% on CyberShake, 13% on Epigenomics, and 11% on Sipht.

## Conclusion

To address the security challenges of scientific workflow systems in the cloud, we propose a FITSW system. The workflow executors in this system are virtual clusters comprised of many VMs, which can improve workflow execution reliability. To detect accidental or malicious errors during the workflow scheduling process, FITSW divides the entire workflow deadline into sub-deadlines for each sub-task. The reliability of the workflow execution is further improved using a new decision mechanism to eliminate unreliable results. Since the workflow environment in a conventional cloud workflow system is static and unchanged, attackers may easily implant a backdoor attack and cause data leakage. To solve this problem, a dynamic task scheduling approach based on resource circulation is introduced. The approach disrupts the attack chain and guarantees that the task executors remain in a clean state. Performance evaluation using the WorkflowSim toolkit shows that the proposed solution achieves an improved scientific workflow intrusion tolerance. The results reveal that FITSW algorithm not only increases the success rate by about 12% but also improves task completion rate by 6.2% and reduces completion time by 15.6%, in comparison to the intrusion tolerant scientific workflow ITSW system.

## Supplemental Information

10.7717/peerj-cs.747/supp-1 Supplemental Information 1 CyberShake is a workflow that assesses the risks in a given area. To characterize the same, it employs the Probabilistic Seismic Hazard Analysis (PSHA) method. This method involves defining a region and then running a finite difference simulation to obtain Strain Green Tensors (SGTs). For each of the ruptures predicted previously, synthetic seismograms are constructed from SGT data. After that, spectral acceleration and probabilistic hazard curves are constructed. To get the results, more than 800,000 jobs were run through the CyberShake workflow. (https://pegasus.isi.edu/workflow_gallery/).

10.7717/peerj-cs.747/supp-2 Supplemental Information 2 Montage is a scientific workflow that may be used to create unique sky mosaics. It takes photos in the FITS (Flexible Image Transport System) format as input. The input photos’ geometry is utilized to determine the output image’s geometry, and then the final mosaic is created. The input images are rotated and re-projected with the same spatial scale. All of the photographs’ background emissions are adjusted to the same level. The final mosaic is formed by combining the corrected and re-projected images. (https://pegasus.isi.edu/workflow_gallery/).

10.7717/peerj-cs.747/supp-3 Supplemental Information 3 The Laser Interferometer Gravity Wave Observatory (LIGO) is a method for detecting gravitational waves emitted during various events, according to Einstein’s general relativity theory. LIGO analyses data from compact binary systems like black holes and binary neutron stars. The data collecting and processing for the LIGO experiments are handled *via* Grid computing technology. This method looks for inspiral signals, which can happen when two compact objects, such as neutron stars or black holes, form binary systems. The items spiral inward over time, resulting in gravitational radiation (https://pegasus.isi.edu/workflow_gallery/).

10.7717/peerj-cs.747/supp-4 Supplemental Information 4 The Epigenomics workflow is a data processing pipeline that automates the execution of genome sequencing activities. After the DNA sequence is generated, it is divided into various chunks that will be operated in parallel. Each chunk’s data is subsequently translated to a file format. After that, noise and contaminating sequences are filtered out, and the sequences are assigned to their proper locations in a genome. It also creates a global map and determines the density of sequence in each place of the genome. At Epigenomic Center, this workflow is utilized to generate histone modification and DNA methylation data (https://pegasus.isi.edu/workflow_gallery/).

10.7717/peerj-cs.747/supp-5 Supplemental Information 5 SIPHT is a program that predicts and annotates gene and bacterial replicon sequences. It involves a number of applications that must be run in the correct order. (https://pegasus.isi.edu/workflow_gallery/).

10.7717/peerj-cs.747/supp-6 Supplemental Information 6 Fault-Intrusion workflow scheduling algorithm Code.

## Additional Information and Declarations

### Data Availability

The following information was supplied regarding data availability: The different scientific workflows that we used in our experiments are available as Supplemental Files.
